# An anatomic consideration of C2 vertebrae artery groove variation for individual screw implantation in axis

**DOI:** 10.1007/s00586-013-2779-4

**Published:** 2013-05-10

**Authors:** Janhua Wang, Hong Xia, Qingshui Ying, Yang Lu, Zenghui Wu, Fzhi Ai, Xiangyang Ma

**Affiliations:** 1 Department of Orthopaedic Surgery, Liu Hua Qiao Hospital (General Military Hospital of Guangzhou Command), 111 Liuhua Road, Yuexiu District, 510010 Guangzhou, China; 2 Department of Radiology, University of Illinois Hospital and Health Sciences System, Chicago, IL 60612 USA

**Keywords:** Safe zone, C2 vertebral artery groove (VAG), C2 pedicle screw, C2 translaminar screw, Individual screw placement

## Abstract

**Study design:**

Retrospective case series.

**Objectives:**

To identify the variation of C2 vertebral artery groove (VAG) based on the thin-slice computed tomography (CT) scan and choose an individual screw placement method to decrease risk of malposition.

**Background:**

C2 pedicle screws can be successful anchors for a variety of cervical disorders. However, variations of VAG may cause malposition and breach when C2 transpedicle screw was inserted. Recognizing the variations of vertebrae artery groove (VAG) in C2 and choosing an individual screw placement method (transpedicle or translaminar) may be helpful for avoiding violation and decreasing the operation risk in upper cervical surgery.

**Methods:**

From January 2009 to December 2010, a total 45 patients with upper cervical disorders underwent 1–mm-thin-slice CT scans along the C2 pedicle direction to obtain the consecutive spectrum of C2 VAG were included in this study. The C2 VAG (types I, II, III, and IV) was subgrouped based on parameter *e* (the vertical distance from the apex of VAG to the upper facet joint surface) and parameter *a* (horizontal distance from the entrance of VAG to the vertebrae canal). Subsequently, individual strategy was used to avoid the VAG violation.

**Results:**

The variations of C2 VAG in these 45 patients include the following: type I 53 (58.9 %), type II 16 (17.8 %) type III 13 (14.4 %), and type IV 8 (8.9 %). Transpedicle screws of C2 were used in types I, III, and IV VAGs (*n* = 74); translaminar screws were inserted in type II subgroup (*n* = 16). Postoperative CT scans showed that there were two pedicle screws violated into the artery groove, and no translaminar screw breached into the vertebrae canal. All the other screws were in right position. None of the 45 patients had severe complications such as spinal cord injury, dura tear, and infection.

**Conclusion:**

Thin-slice CT scan along the C2 pedicle direction to analysis the variations of C2 VAG can help choose an individual screw placement method (transpedicle or translaminar) with minimal complication for C2 screw fixation.

## Introduction

Segmental screw fixation of the upper cervical spine using transpedicle screws at C1/C2 and/or laminar screws at C2 are becoming increasingly popular [1–3]. However, there are potential risks of screw malposition such as cortical breaches while placing C1 or C2 pedicle screws. Neo et al. [4] reported a breach rate of 29 % with the majority of violations occurring laterally into vertebrae artery groove (VAG) in the C2 pedicle screw placement. Variation of VAG may play a role for malposition and breach when C2 transpedicle screw was inserted. Identifying the variation of VAG and choosing an individual screw placement method (transpedicle or translaminar) may be helpful for avoiding violation and decreasing the complications in upper cervical surgery.

## Materials and methods

### Patients’ preoperative planning by fine-cut computed tomography (CT) scan analysis and operations

#### Preoperative planning

**Fig. 1:**
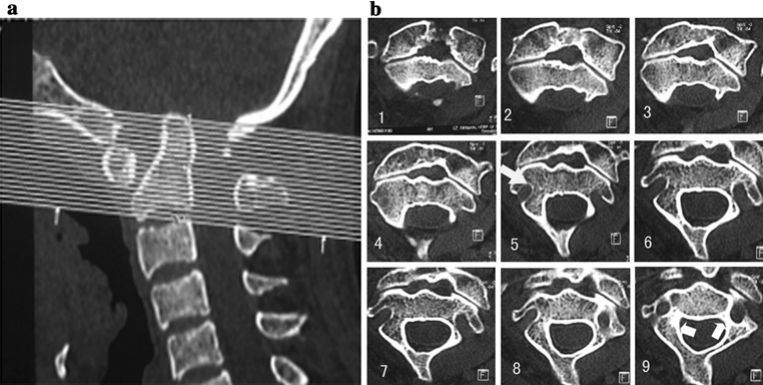
**a** 1-mm-thin-slice consecutive CT scan of C2 was carried out along the C2 pedicle direction (about 15°–20° superiorly). **b** In the consecutive axial thin-slice CT of C2, *white arrows* in the 5th slice show the apex of VGA, and then, parameter *e* could be calculated by the number of slices easily; *white arrows* in the 9th slice show the distance from the entrance of VGA to the vertebrae canal, and then, parameter *a* could be measured in this slice)

**Fig. 2:**
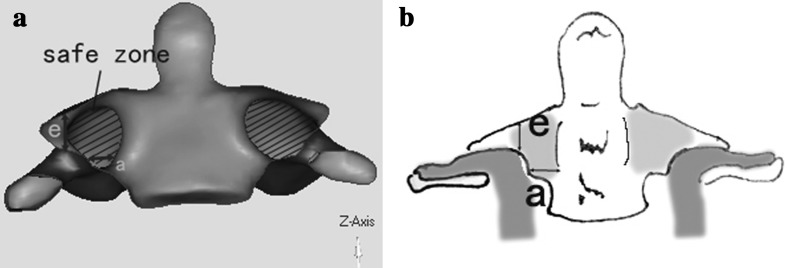
**a** reconstructed image of C2 CT scan showing a safe zone for pedicle screw placement boarded by parameter *a* and *e*. **b** VAG model figure could be reconstructed easily based on parameter *a* and parameter *e* measured on the consecutive CT scan of C2

From January 2009 to December 2010, there were total 45 consecutive patients with upper cervical disorders received preoperative thin-slice (1 mm thickness) CT scans along C2 pedicle direction to obtain the consecutive picture of C2 VAG (Fig. 1a, b), and then, reconstruction image of C2 was established with mimic 10.0 software (Fig. 2a).

In the reconstructed images of the C2 (Fig. 2a), we could found a “safe zone” for screw placement in the inferior and superior room from the VGA, which could be defined by parameter *a* (the vertical distance from the apex of VAG to the upper facet joint surface) and parameter *e* (horizontal distance from the entrance of VAG to the vertebrae canal). For more easy use of “safe zone” on the consecutive CT scan pictures, parameter *e* could be measured by the number of scan layer for the VAG apex just show, and parameter *a* could be measured by the diameters of the C2 pedicle on the VAG entrance slice of CT scan (Fig. 1a, b); then, a model figure of C2 could be easily established based on parameter *e* and parameter *a* (Fig. 2b).

**Fig. 3:**
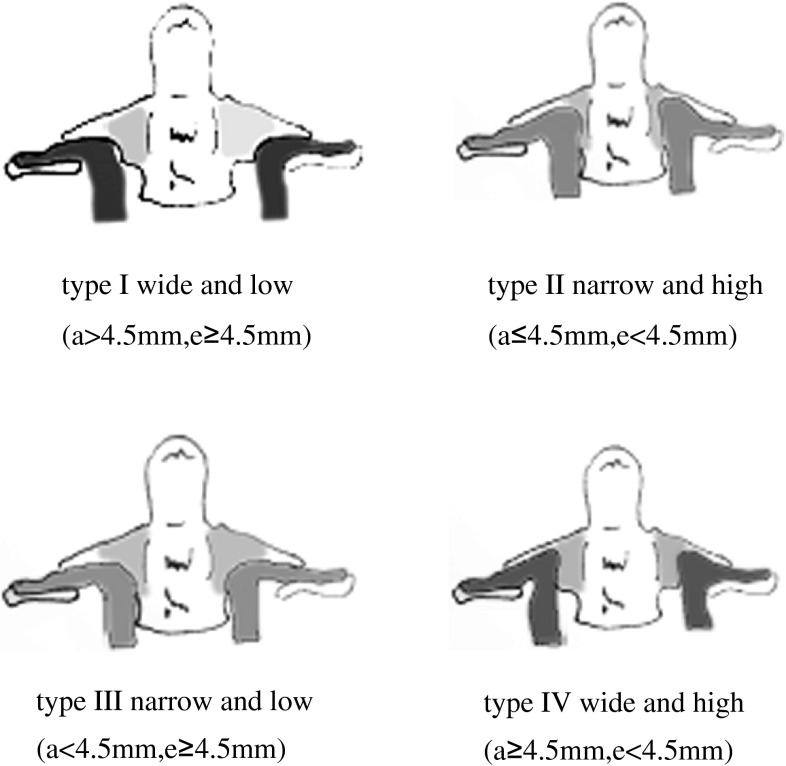
Classification of C2 VAG

The larger the area of this safe zone, the safer for screw implantation through the axis pedicle (Fig. 3). In consideration of the frequently used 3.5-mm screw, we divide the C2 VAG into four subgroups based on parameter *a* and *e* as showed in (Table 1; Fig. 3). Parameter *e* was defined low or high with cutoff value of 4.5 mm, and parameter *a* was defined narrow or wide with cutoff value of 4.5 mm, respectively. Therefore, the C2 VAG could be divided into four subgroups (Table 1; Fig. 3).

**Table 1:** Classification for C2 VAG based on thin-slice CT scan spectrum

Subgroup		Criteria	Indication or C2 screw placement
Type I	Wide and low	*a* > 4.5 mm, *e* ≥ 4.5 mm	Pedicle screw
Type II	Narrow and high	*a* ≤ 4.5 mm, *e* < 4.5 mm	Lamina screw
Type III	Narrow and low	*a* < 4.5 mm, *e* ≥ 4.5 mm	Pedicle screw
Type IV	Wide and high	*a* ≥ 4.5 mm, *e* < 4.5 mm	Pedicle screw

**Fig. 4:**
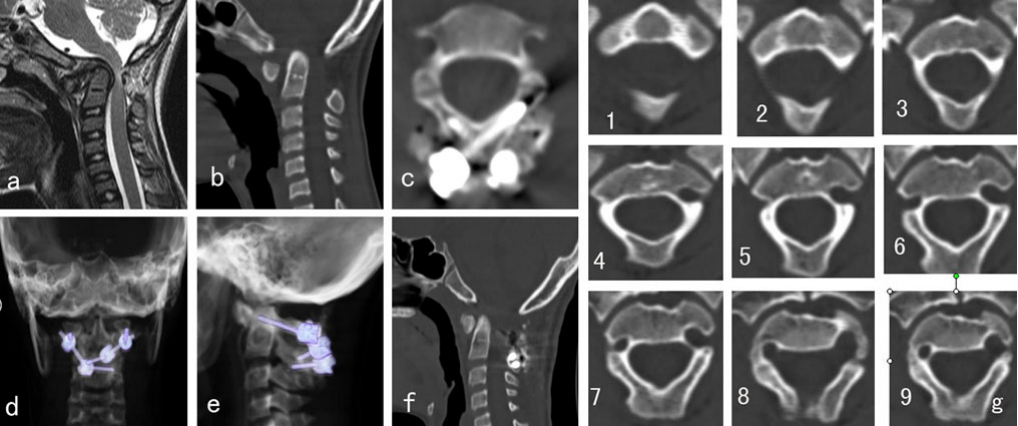
A 16-year-old female patient, **a**, **b** CT and MR show atlantoaxial dislocation, the spine medulla was compressed by the dens, **g** the consecutive C2 CT scan spectrum shows that the VAG was type II in both sides; **c–e** the patient underwent posterior instrumentation with lamina screws on both sides of C2, **f** 3-month postoperative CT scan showed atlantoaxial dislocation was reduced appropriately

In type II VAG, the VAG is high ride and the entrance of VAG is very close to the vertebrae canal; thus, the “safe zone” for pedicle screw placement is poor for a 3.5-mm screw. We then choose translaminar screw for patients with type II VAG (Fig. 4). While patients with other types of VAG (including types I, III, or IV) received transpedicle screw fixation, all consecutive patients underwent segmental screw fixation at C1 and/or C2 or as a part of an occipitocervical reconstruction by the same spine surgeon.

According the value of parameter *a* and *e*, the C2 groove was classified into four types: type I wide and low, type II narrow and high, type III narrow and low, and type IV wide and high.

#### Operation technique

Operations were carried out under lateral C arm fluoroscopic guidance, without intraoperative navigation. The same technique was used in all cases. C1 transpedicle or lateral mass screws and C2 transpedicle screws were placed under fluoroscopic guidance, whereas C2 lamina screws were placed using a free-hand technique. The entry point of C2 pedicle screws was 3 to 7 mm caudal to the C1–C2 joint and 3–4 mm lateral to the medial border of the pars. And the screws were directed approximately 30° medial on the axial plane and toward the anterior–superior end of the superior articular process or just below observed by lateral fluoroscopy. C2 laminar screws were placed at the spinolaminar junction and directed along the contralateral laminar surface. Once the entry point and trajectory were determined, a 3.0-mm drill was used to create the screw tract. A ball-tipped probe was then used to palpate the tract to ensure that no cortical violations were detected. The screw tract was then prepared using the appropriately sized tap and an appropriately sized screw was inserted.

#### Postoperative evaluation

All patients underwent plain X-rays and thin-slice CT scans to define the position of the screws and the extent of reduction 1 week after the surgery. All the investigators had access to review coronal and sagittal thin-slice CT images in each patient. Fusion was considered successful when the postoperative follow-up CT scan showed a bone bridge formation and a dynamic X-ray showed a stable reduction in the dislocation without failure of the implantation. If a breach was observed, the breach was classified as <2, <4, or >4 mm according to the maximum value of cortical violation. Two physicians evaluated each image independently and reached consensus on interpretation. Magnetic resonance imaging (MRI) was performed to assess the extent of decompression of the spinal cord. Dynamic X-rays and CT scans with reconstruction view were performed during follow-up visits at 3, 6, and 12 months after surgery to check the position of the implants and bone fusion. All the patients were followed up for 13–28 months (mean 14 ± 3 months). The JOA score was used to evaluated the spinal function and improvement 3 months after surgery.

### Statistical analysis

A paired Student’s test was performed for pre- and postoperative result. The level of significance was set at *p* < 0.05. Statistical analyses were performed using SPSS 13.0 for windows.

## Result

### Patient and demographic information

Total 45 consecutive patients were included in this study, consisting of 25 men and 20 women with a mean age of 43 years (range from 15 to 63 years). 41 patients had segmental screw fixation of C1–C2 alone: 15 patients with os odontoideum associated with atlantoaxial dislocation, three with rheumatoid arthritis with evidence of instability or impaction, 23 with traumatic atlantoaxial dislocation. The other four patients underwent C2 screw placement as a part of an occipitocervical reconstruction: two patients with traumatic C1 lateral mass fracture and two had atlas tuberculosis.

#### Screw implantation and clinical result

According to the parameter *a* and parameter *e* measured in the thin-slice CT scan spectrum of C2, the subgroup of C2 groove in these 45 patients includes the following: type I 53 (58.9 %), type II 16 (17.8 %) type III 13 (14.4 %), and type IV 8 (8.9 %). In the types I, III, and IV grooves, the pedicle size was confirmed to be large enough to accommodate a 3.5-mm screw; therefore, 73 transpedicle screws of C2 were used. The other 17 C2 VAG were recognized as type II subgroup because the groove shows a high ride shape, and the diameter of axis pedicle in the last layer is very small (lesser than 4.5 mm).The “safe zone” for screw implant is poor, while the thickness of lamina is enough for 3.5-mm screw placement; therefore, 17 translaminar C2 screws were inserted.

**Fig. 5:**
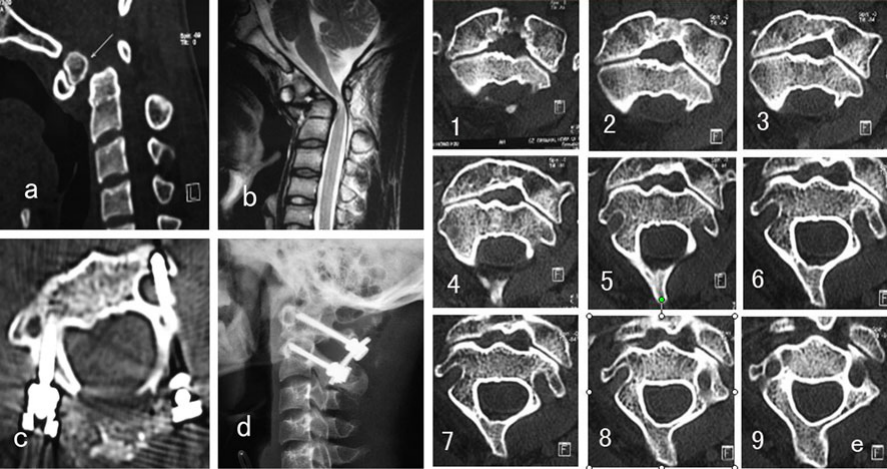
A 43-year-old male patient, showing **a** atlantoaxial dislocation associated with os odontoideum, **b** atlantoaxial dislocation and compression of medullar in the upper cervical, **e** the consecutive CT scan spectrum shows that the C2 VGA was type III (*left side*) and type I (*right side*), the patient underwent transpedicle screw placement on both side of C2, (**c**, **d**) show the pedicle screw is in *right side* is in good position, but the *left* screw violated into the VAG

The postoperative CT scan shows that there are two pedicle screws violated into the artery groove (Figs. 4, 5), (1 < 2 mm, 1 < 4 mm), and no translaminar screw breaches into the vertebrae canal. All the other screw was placed in right position. None of the 45 patients had any severe complication such as spinal cord injury, dura tear, or infection. X-ray showed all the atlantoaxial dislocation was reduced appropriately. Solid fusion was achieved in 44 patients at 5 to 10 months after surgery. One patient had loosing of screw in the Atlanta and no union at 4-month postoperative follow-up. Subsequently, a revision operation with occipital–cervical instrumentation was performed, and the patient gained solid fusion 6 months later. Postoperation CT scans showed the mean atlantodens index (ADI) changed from 8.43 ± 2.33 to 2.55 ± 1.32 (*p* < 0.01 %), MRI showed that the mean cervicomedullary angle (CMA) changed from 136.3° ± 8.8° to 159.5° ± 9.22° (*p* < 0.01 %). All patients had different extents of improvement on the spinal function, and the average JOA improve from 9.5 ± 1.3 to 14.8 ± 1.6 (*p* < 0.01 %).

## Discussion

C2 pedicle screw can be used as a successful anchor for correction and fusion of a variety of atlantoaxial and occipitocervical problems [5–7]. Biomechanical studies have shown the stability of C2 pedicle screws in a highly mobile region [8, 9]. Pedicle screws could offer adequate fixation of the axis and comparable fusion rates compared with those obtained with transarticular screws. However, placement of these screws is technically demanding and places the vertebral arteries and nerve roots at risk of damage in circumstances of cortical breach [10].

Stulik et al. [11] reported 5.4 % of screws malpositioned placed in C2. Ondra et al. [12] reported the result of their 150 C2 pedicle screws placed in 79 patients, and there were 8 VA foraminal breaches on postoperative CT scans. Yeom et al. [13] analyzed the incidence of cortical breaches for VAG in C2 pedicle screws using postoperative fine-cut CT scans and CT angiography with multi planar and three-dimensional reconstructions. They reported a higher VAG violations rates (20 %). They speculated that the frequency of VAG violation may be underestimated by many authors due to inaccurate evaluation methods. As the intraoperative lateral C arm fluoroscopic monitoring and postoperative radiographs may not be enough for assistant, avoid this kind of risk in C2 transpedicle screw placement. Therefore, it is necessary to investigate the anatomic character of C2 VAG furtherly and set up an individual surgery strategy may be helpful for decrease the risk of VAG violation.

It has been reported that anatomic variations of C2 VAG found on preoperative imaging have impact on surgical planning [14]. However, there is no well-accepted guidelines on which radiographic parameters can predict risks for cortical breach with C2 pedicle screw placement. Preoperative CT has been shown to improve surgeon ability to detect important differences in vertebral anatomy [15]. Hassan et al. [16] reported that the C2 pedicle screws placement risk could be judged on the size of pedicle shown on presurgical thin-slice CT scan, as the diameter of C2 pedicle less than 6 mm was associated with a nearly twofold higher risk of cortical breach than the group more than 6 mm (37 vs 21 %); therefore, measurement of the pedicle diameter on CT scan could act as an useful parameter for evaluation of the risks of screw placement. However, measurement of the diameter of C2 pedicle varies among different authors, usually with different methods and on different slices of the CT scan.

Computer-assisted three-dimensional reconstruction is a good way for the evaluation of C2 VGA; however, it is time-consuming and complicated. We are seeking a simple and useful way for C2 VGA evaluation. By using consecutive thin-slice CT scan (1 mm thickness), a kind of CT scan spectrum of C2 VAG could be easily obtained. Compared with the single-slice image of C2 pedicle, the consecutive CT scan spectrum could provide us a kind of holography of the C2 VAG with more integrated and rich information. Through this C2 VAG CT spectrum, we could reconstruct a kind of model figure of C2 VAG easily just like the three-dimensional reconstruction CT image based on parameter *a* and parameter *e,* and a “safe zone” for pedicle screw placement could be easily found on the model figure of C2 too.

When inserting the screw through C2 pedicle, the room surrounded by the “*a* and *e*” in the coronal section of pedicle will provide a “safe zone” for screws placement. The larger the *a*/*e* value is, the safer for pedicle screw implantation. When the *a*/*e* lesser than 4.5/4.5 mm (type II subgroup), it is difficult and dangerous to place a 3.5 mm screw, as violation could happen easily.

For the subgroup of types I, III, IV, the “safe zone” of “*a* and *e*” is bigger than 4.5 × 4.5 mm, which could provide a relative safe room for pedicle placement. However, in the subgroup type II, the “safe zone” of “*a* and *e*” is lesser than 4.5 × 4.5 mm, which should be regard as contradiction for pedicle screw placement. Therefore, an alternative method of C2 translaminar screw should be recommended. The major advantage of C2 translaminar screw is the elimination of the potential risk of arterial injury by placing screws only within the posterior column [17]. In our 45 consecutive patients, there were 74 transpedicle screws and 16 translaminar screws used according the above strategy rules. The postoperative CT scan shows that there were two pedicle screws violated into the artery groove (2.7 %), and no translaminar screw breached into the vertebrae canal, which show a smaller breach rate than the lecture reported by Yeom and other authors [11–13]. Yeom et al. claimed that the risk of pedicle violation cannot be completely avoided, even with careful preoperative planning and intraoperative C arm fluoroscopic imaging. We think that choosing an individual screw placement method (transpedicle or translaminar) based on presurgical thin-slice CT analysis of C2 VAG variations could provide an useful personalized strategy for C2 screw fixation, diminishing complications and risks, and lower the violation rates.
